# Supplementary material for: Hepatitis A Seroprevalence Among HIV-Exposed and Unexposed Pediatric Populations in South Africa
Source: Vaccines (Basel). 2024 Nov 13;12(11):1276. doi: 10.3390/vaccines12111276 (PMC11598911; doi:10.3390/vaccines12111276)
Supplement: Supplementary file 1 [file vaccines-12-01276-s001.zip › Supplementary Table S1_Amponsah-Dacosta.pdf]

Supplementary Table S1. Participants' socio-demographic characteristics stratified by perinatal HIV exposure and infection status

	**Total N=513 (%)**	**HIV[1] n=19 (%)**	**HEU[2] n=74 (%)**	**HIV-E[3] n=8 (%)**	**HUU[4] n=412 (%)**
**Sex**					
Male	277 (54.0%)	10 (52.6%)	44 (59.5%)	5 (62.5%)	218 (52.9%)
Female	236 (46.0%)	9 (47.4%)	30 (40.5%)	3 (37.5%)	194 (47.1%)
**Age**					
Median Age (IQR) months	10 (4, 25)	19 (8, 56)	10 (4, 18)	7 (4, 10)	10 (4, 26)
<1 year	276 (53.8%)	6 (31.6%)	44 (59.5%)	6 (75.0%)	220 (53.4%)
1 – 4 years	177 (34.5%)	8 (42.1%)	24 (32.4%)	1 (12.5%)	144 (35.0%)
5 – 12 years	60 (11.7%)	5 (26.3%)	6 (8.1%)	1 (12.5%)	48 (11.7%)
**Ethnicity**					
Black	337 (65.7%)	17 (89.5%)	73 (98.6%)	6 (75.0%)	241 (58.5%)
Coloured/Mixed ancestry	164 (32.0%)	2 (10.5%)	1 (1.4%)	—	161 (39.1%)
Other ethnicity	1 (0.2%)	—	—	—	1 (0.2%)
Unknown[5]	11 (2.1%)	—	—	2 (25.0%)	9 (2.2%)
**Crèche attendance[6]**					
Yes	129 (25.1%)	9 (47.4%)	16 (21.6%)	3 (37.5%)	101 (24.5%)
No	380 (74.1%)	10 (52.6%)	57 (77.0%)	5 (62.5%)	308 (74.8%)
Unknown[5]	4 (0.8%)	—	1 (1.4%)	—	3 (0.7%)
**Feeding at ≤4 months**					
Breast milk	390 (76.0%)	17 (89.5%)	46 (62.2%)	5 (62.5%)	322 (78.2%)
Breast milk & formula	108 (21.1%)	2 (10.5%)	27 (36.5%)	3 (37.5%)	76 (18.4%)
Formula (never breastfed)	14 (2.7%)	—	1 (1.4%)	—	13 (3.2%)
Unknown[5]	1 (0.2%)	—	—	—	1 (0.2%)
**Feeding at >4 months**					
Breast milk	9 (1.8%)	—	1 (1.4%)	—	8 (1.9%)
Breast milk & formula	468 (91.2%)	15 (78.9%)	65 (87.8%)	8 (100%)	380 (92.2%)
Formula (never breastfed)	11 (2.1%)	—	2 (2.7%)	—	9 (2.2%)
Unknown[5]	25 (4.9%)	4 (21.1%)	6 (8.1%)	—	15 (3.6%)

[1]Children living with HIV; [2]HIV-exposed uninfected; [3]HIV-exposed but child infection status unknown; [4]HIV-unexposed uninfected; [5]missing data; [6]preschool
