# Supplementary material for: Hepatitis A Seroprevalence Among HIV-Exposed and Unexposed Pediatric Populations in South Africa
Source: Vaccines (Basel). 2024 Nov 13;12(11):1276. doi: 10.3390/vaccines12111276 (PMC11598911; doi:10.3390/vaccines12111276)
Supplement: Supplementary file 1 [file vaccines-12-01276-s001.zip › Supplementary Table S2_Amponsah-Dacosta.pdf]

Supplementary Table S2. Maternal socio- demographic and economic characteristics stratified by participants' HIV exposure and infection status

	Total N=513 (%)	HIV[1] n=19 (%)	HEU[2] n=74 (%)	HIV-E[3] n=8 (%)	HUU[4] n=412 (%)
**Age (years)**					
Median Age (IQR)	29 (25, 34)	29 (26, 34)	32 (26, 34)	31 (27, 37)	28 (24, 34)
Teen (15 – 19)	33 (6.4%)	—	4 (5.4%)	—	29 (7.0%)
Young (20 – 21)	32 (6.2%)	—	3 (4.1%)	1 (12.5%)	28 (6.8%)
Adult (22 – 51)	448 (87.3%)	19 (100%)	67 (90.5%)	7 (87.5%)	355 (86.2%)
**Education level**					
Primary	20 (3.9%)	—	4 (5.4%)	1 (12.5%)	15 (3.6%)
Secondary	460 (89.7%)	19 (100%)	67 (90.5%)	5 (62.5%)	369 (89.6%)
Highest basic school grade unknown[5]	18 (3.5%)	—	2 (2.7%)	2 (25.0%)	14 (3.4%)
Tertiary	15 (2.9%)	—	1 (1.4%)	—	14 (3.4%)
**Type of dwelling**					
Bricks	298 (58.1%)	7 (36.8%)	25 (33.8%)	2 (25.0%)	264 (64.1%)
Shack (tin/zinc/iron sheeting)	196 (38.2%)	12 (63.2%)	46 (62.2%)	6 (75.0%)	132 (32.0%)
Wood or Wendy house	16 (3.1%)	—	3 (4.1%)	—	13 (3.2%)
Unknown[6]	3 (0.6%)	—	—	—	3 (0.7%)
**Water source**					
In-door tap	239 (46.6%)	5 (26.3%)	18 (24.3%)	1 (12.5%)	214 (51.9%)
Outdoor communal tap	110 (21.4%)	3 (15.8%)	26 (35.1%)	2 (25.0%)	80 (19.4%)
Borehole	164 (32.0%)	11 (57.9%)	30 (40.5%)	5 (62.5%)	118 (28.6%)
**Type of toilet**					
Flush toilet	435 (84.8%)	16 (84.2%)	54 (73.0%)	6 (75.0%)	359 (87.1%)
Bucket	8 (1.6%)	—	2 (2.7%)	1 (12.5%)	5 (1.2%)
Pit latrine	67 (13.1%)	3 (15.8%)	17 (23.0%)	1 (12.5%)	46 (11.2%)
Unknown[6]	3 (0.6%)	—	1 (1.4%)	—	2 (0.5%)
**Socio-economic quartiles[7]**					
Low	104 (20.3%)	4 (21.1%)	20 (27.0%)	2 (25.0%)	78 (18.9%)
Lower-middle	34 (6.6%)	—	1 (1.4%)	1 (12.5%)	32 (7.8%)
Upper-middle	247 (48.1%)	11 (57.9%)	36 (48.6%)	3 (37.5%)	197 (47.8%)
High	64 (12.5%)	2 (10.5%)	10 (13.5%)	—	52 (12.6%)
Unknown[6]	64 (12.5%)	2 (10.5%)	7 (9.5%)	2 (25.0%)	53 (12.9%)

[1]Children living with HIV; [2]HIV-exposed uninfected; [3]HIV-exposed but child infection status unknown; [4]HIV-unexposed uninfected; [5]missing data; [5]participants indicated having received a basic education (primary or secondary) however highest grade attained was not recorded; [6]missing data; [7]socio-economic status was categorized into quartiles based on a validated weighted composite score that included maternal asset ownership, employment, and education
