# Supplementary material for: Hepatitis A Seroprevalence Among HIV-Exposed and Unexposed Pediatric Populations in South Africa
Source: Vaccines (Basel). 2024 Nov 13;12(11):1276. doi: 10.3390/vaccines12111276 (PMC11598911; doi:10.3390/vaccines12111276)
Supplement: Supplementary file 1 [file vaccines-12-01276-s001.zip › Supplementary Table S3_Amponsah-Dacosta.pdf]

Supplementary Table S3. *Univariable logistic regression analysis of socio- demographic and economic factors associated with hepatitis A seropositivity*

**Socio- demographic & economic variables**	**Hepatitis A Seropositivity, *n***	**[1]Hepatitis A seropositivity [OR (95%CI)]**
**Age (years)**	**208**	
<1 (reference)	144	—
1 – 4	36	0.23 (0.14-0.36)
5 – 12	28	0.80 (0.45-1.40)
**Sex**	**208**	
Female (reference)	102	—
Male	106	0.81 (0.57-1.16)
**Maternal age (years)**	**208**	
15 – 19 (reference)	12	—
20 – 21	13	1.20 (0.44-3.29)
22 – 51	183	1.21 (0.60-2.60)
**Education level**	**208**	
Primary (reference)	7	—
Secondary	187	1.27 (0.51-3.44)
Tertiary	6	1.23 (0.30-5.00)
Highest basic school grade unknown	8	1.48 (0.40-5.67)
**Socio-economic quartiles**	**208**	
Unknown (reference)	26	—
Low	38	0.84 (0.44-1.60)
Lower middle	15	1.15 (0.49-2.68)
Upper middle	101	1.02 (0.58-1.78)
High	28	1.13 (0.56-2.30)
**Maternal HIV exposure**	**208**	
Negative	163	—
Positive	45	1.23 (0.79-1.90)
**Type of dwelling**	**208**	
Bricks (reference)	121	—
Unknown	1	0.73 (0.03-7.71)
Other	121	0.33 (0.07-1.07)
Shack (tin/zinc/iron sheeting)	83	1.07 (0.74-1.55)
Other	3	0.34 (0.08-1.07)
**Water source**		
Borehole (reference)	75	—
Indoor tap	96	0.80 (0.53-1.19)
Outdoor/communal tap	37	0.60 (0.36-1.00)
**Type of toilet**	**208**	
Bucket system (reference)	2	—
Flush toilet	177	2.06 (0.47-14.16)
Unknown	2	6.00 (0.38-182.92)
Pit latrine	27	2.03 (0.43-14.53)
**Feeding at ≤4 months**	**208**	
Breast milk (reference)	158	—
Breast milk & formula	41	0.89 (0.58-1.38)
Formula (never breastfed)	9	2.64 (0.89-8.74)
Unknown	0	[2]NC
**Feeding at >4 months**	**208**	
Breast milk (reference)	3	—
Breast milk & formula	188	1.34 (0.35-6.42)
Formula (never breast fed)	5	1.66 (0.27-1.38)
Unknown	12	1.84 (0.39-0.33)

[1]Reference p-value =0.05; [2]NC = Not computed
